# Long-Term Outcome of Splanchnic Vein Thrombosis in Cirrhosis

**DOI:** 10.1038/s41424-018-0043-2

**Published:** 2018-08-15

**Authors:** Marco Senzolo, Nicoletta Riva, Francesco Dentali, Kryssia Rodriguez-Castro, Maria Teresa Sartori, Soo-Mee Bang, Ida Martinelli, Sam Schulman, Adriano Alatri, Jan Beyer-Westendorf, Matteo Nicola Dario Di Minno, Walter Ageno, for the IRSVT study investigators

**Affiliations:** 1 Multivisceral Transplant Unit, University Hospital of Padua, Padua, Italy; 2 Department of Clinical and Experimental Medicine, University of Insubria, Varese, Italy; 3 Clinical Medicine I, Department of Medicine, Padua University Hospital, Padua, Italy; 4 Department of Internal Medicine, Seoul National University Bundang Hospital, Seongnam, Republic of Korea; 5 A. Bianchi Bonomi Hemophilia and Thrombosis Center, Fondazione IRCCS Ca’ Granda, Ospedale Maggiore Policlinico, Milan, Italy; 6 Department of Medicine, McMaster University, Hamilton, Canada; 7 Hemostasis and Thrombosis Center, A.O. Istituti Ospitalieri di Cremona, Cremona, Italy; 8 Thrombosis Research, Department of Medicine, 1st Division of Hematology, Dresden University Clinic, Dresden, Germany; 9 Department of Advanced Biomedical Sciences, Division of Cardiology, Federico II University, Naples, Italy

## Abstract

**Introduction:**

Little is known about the long-term outcome of cirrhotic patients with splanchnic vein thrombosis (SVT). This prospective cohort study aimed to describe the clinical presentation, bleeding incidence, thrombotic events, and mortality in patients with SVT associated with cirrhosis.

**Methods:**

Among 604 consecutive patients with SVT enrolled over 2 years, 149 had cirrhosis. Major bleeding, thrombotic events, and all-cause mortality were recorded during a 2-year follow-up. In a subgroup, the degree of recanalization with or without anticoagulation therapy, and the correlation between clinical events and liver disease severity were also investigated.

**Results:**

The most common thrombosis sites were the portal (88%) and mesenteric veins (34%). At presentation, 50% of patients were asymptomatic. Anticoagulation was administered to 92/149 patients for a median of 6.5 months. Vessel recanalization was documented in 47/98 patients with a radiological follow-up. Anticoagulation was associated with a 3.33-fold higher of recanalization rate, and a lower recurrent thrombosis rate, while patients with and without anticoagulation experienced a similar rate of major bleeding episodes. Mortality rates were 6.8 per 100 patient-years for patients with thrombosis completely or partially resolving during the follow-up, and 15.4 per 100 patient-years for those with stable or progressing thrombosis. An impact of SVT on survival was only apparent in patients with more advanced liver disease (Child–Pugh B-C).

**Conclusions:**

Patients with SVT and cirrhosis have a substantial long-term risk of recurrent thrombotic events, which is reduced by anticoagulation therapy without any increase in bleeding risk. Anticoagulation can improve the likelihood of vessel recanalization, and is associated with a lower risk of death for decompensated patients.

## Introduction

Splanchnic vein thrombosis (SVT), and portal vein thrombosis (PVT) in particular, is a common complication in patients with liver cirrhosis, and poses a number of clinical challenges. The prevalence of PVT in cirrhotic patients varies from 5 to 26%^1–3^, with a 10% incidence at 1 year;^4^ the incidence of non-portal SVT has not been reported.

The influence of PVT and SVT on the natural history of cirrhosis is still under debate. Conflicting results have been reported in the literature due to different populations being studied, and due to a lack of large, dedicated prospective studies taking both liver disease severity and the characteristics of the cases of thrombosis into account^5^.

The role of anticoagulant treatment in cirrhosis-related PVT, with and without involvement of the superior mesenteric and splenic veins, has been examined in few small studies (on 19–26 patients). Most of the patients considered in these analyses had a partial/incomplete thrombosis, and the complete recanalization rates varied between 40 and 75%^6–8^. A few publications have described the natural history of untreated PVT in patients with cirrhosis, but the cases were heterogeneous in terms of liver disease severity, the incidence of thrombus progression or stabilization, and the cases of liver decompensation and death^5^.

We previously reported the results of a prospective cohort study on patients with SVT^9^. Here, we describe a subgroup analysis conducted on the patients with cirrhosis-associated SVT, our aim being to assess the degree of portal vein recanalization with and without anticoagulation treatment, and the correlation between long-term clinical events (including major bleeding episodes, thrombotic events, and mortality) and the severity of the associated liver disease.

## Methods

### Study design

A multicenter, international, prospective cohort study, the International Registry on Splanchnic Vein Thrombosis (IRSVT), promoted by the International Society on Thrombosis and Hemostasis (ISTH), was conducted from 2 May 2008 to 30 January 2012 on consecutive patients with SVT objectively diagnosed no more than 6 months prior to their inclusion in the trial. The protocol was approved by the institutional review board or ethical committee of each participating center, and written informed consent was obtained from patients, where necessary (informed consent is not required for observational studies in some countries). Participants did not receive any financial compensation. Data were collected, maintained, and analyzed by the Research Center on Thromboembolic Diseases and Antithrombotic Drugs at the University of Insubria, Varese, Italy.

The diagnosis of SVT was accepted if confirmed by Doppler ultrasonography, computed tomography, magnetic resonance imaging, or angiography, or during laparoscopic or abdominal surgery. Neoplastic thrombi of the portal vein in the patients with hepatocellular carcinoma (HCC) were diagnosed according to the standard radiological criteria^10^, and patients with HCC and a demonstrated tumor thrombus were excluded. Treatment decisions were left entirely to the discretion of attending clinicians, and no therapeutic algorithms were provided. Patients were monitored with regular visits for 2 years, and all participating centers were invited to provide follow-up information on the study outcomes at least every 6 months. The follow-up was terminated on 30 January 2014.

Investigators recorded the data on a computer-based case report form, and submitted the forms to a centralized coordinating center using a secure website. The coordinating center at the University of Insubria used multiple data quality-control procedures to optimize the data quality. Data were monitored routinely to check for inconsistencies or errors, where necessary, and queries were sent to the local investigators at each participating center. All reported clinical outcomes were accepted by a central review committee.

For the cohort of cirrhotic patients, a diagnosis of cirrhosis was established from their medical history, liver radiology, and liver biopsy or noninvasive assessment of fibrosis, where available. For the purpose of the present analysis, patients with Budd–Chiari syndrome were excluded.

### Study variables

The following baseline data were collected at the time of patients’ inclusion in the study: demographic characteristics, family or personal history of venous thromboembolism, inherited and acquired thrombophilic risk factors and markers of Philadelphia-negative myeloproliferative neoplasms, use of hormone therapy, and other potential risk factors (e.g., cancer, intra-abdominal inflammatory conditions, hematological disorders, and recent abdominal surgery).

Patients’ clinical characteristics were recorded, including the local extent of the SVT at the time of its diagnosis, biochemical tests, and liver disease severity assessed in terms of Child–Pugh class and Model for End-Stage Liver Disease (MELD) score, presence of ascites or varices, use of unfractionated heparin or low-molecular-weight heparin, use of vitamin K antagonists, and use of other antithrombotic and/or thrombolytic drugs, or any other treatments.

During the follow-up, information was collected during antithrombotic treatments, and on clinical outcomes (major bleeding episodes, vascular events, and mortality). Major bleeding was defined as fatal bleeding, bleeding necessitating surgery, bleeding in a critical organ (intracranial or intraspinal, retroperitoneal, or intraocular resulting in visual impairment), overt bleeding associated with a drop in hemoglobin levels of 2 g/dL or more (to convert to grams per liter, multiply by 10), bleeding requiring the transfusion of 2 U or more of red blood cells, or bleeding leading to hospitalization. Major bleeding episodes were classified according to whether they developed from portal hypertensive complications or other sources. Thrombotic events included recurrent SVT (defined as thrombus extending to or occurring in a previously patent segment), symptomatic venous thromboembolism at other sites diagnosed by appropriate imaging tests depending on the site, arterial thrombosis (acute coronary syndromes, acute ischemic stroke, transient ischemic attack, and acute peripheral arterial disease) diagnosed according to standard criteria, and mesenteric infarction revealed on a pathology specimen.

Imaging tests were performed during the follow-up, not according to a strict protocol, but at the discretion of the attending physicians. In a post-hoc analysis, SVT progression was investigated, and if recanalization was observed, the time of this imaging finding was considered as the time of recanalization. This approach had already been used in a previous study dealing with venous thromboembolism at unusual sites^11^.

### Statistical analysis

The population’s baseline characteristics are reported using descriptive statistics: continuous variables are expressed as means (standard deviations [SD]) or as medians (interquartile ranges [IQR]) depending on the data distribution; and categorical data as counts and percentages. Continuous variables were compared using Student’s *t*-test or the Mann–Whitney *U* test, and categorical variables using the chi-square or Fisher’s exact tests, as appropriate.

The primary analysis was performed up to the first confirmed clinical outcome (major bleeding episode or thrombotic event). The number of events is expressed as an incidence rate (with a 95% confidence interval [CI]), calculated as the number of events per 100 patient-years of observation. Overall survival rates were assessed with the Kaplan–Meier method. The incidence rate was also calculated for major bleeding episodes and thrombotic events occurring on anticoagulant treatment, and off treatment (distinguishing between patients who had discontinued the treatment and those who had never been treated).

To explore the role of potential predictors of major bleeding and thrombotic events, different multivariable Cox’s proportional hazards regression models were analyzed, using backwards stepwise removal of the variables (with levels of *P* < 0.05 for inclusion and *P* > 0.10 for exclusion, respectively), and stratifying by center to account for any heterogeneity of within-center bleeding and thrombotic risks. The analysis started with the following variables: age, male sex, Asian ethnicity, personal history of venous thromboembolism, incidentally detected SVT, gastrointestinal bleeding at onset, ascites, esophageal varices, solid cancer, anemia (hemoglobin ≤10 g/dL), thrombocytopenia (platelet count ≤50 × 10^3^/mm^3^), creatinine >1.5 mg/dL, and Child–Pugh class, with anticoagulant treatment defined as the time-dependent variable. The variables with a significance level of *P* < 0.05 on multivariable analysis were assumed to be associated with the outcome of interest. The same method was used to investigate potential predictors of mortality and of vessel recanalization, again stratifying by center to account for possible heterogeneity of the within-center likelihood of these events. The analysis started with the following variables: age, male sex, Asian ethnicity, personal history of venous thromboembolism, incidentally detected SVT, gastrointestinal bleeding at onset, ascites, esophageal varices, PVT, solid cancer, anemia (hemoglobin ≤10 g/dL), creatinine >1.5 mg/dL, Child–Pugh class, MELD score, and anticoagulant treatment (defined as the use of anticoagulation medication begun during the acute phase). Variables with a *P* value <0.05 on multivariable analysis were considered to be associated with the outcome of interest. The STATA SE 12.0 software (StataCorp LP, College Station, TX, USA) was used for the statistical management of the data.

## Results

In all, 604 patients seen at 31 centers in 11 countries were prospectively enrolled in the main study. The list of participating centers is provided in the Supplementary material.

For the present subgroup analysis, we identified 167 patients with cirrhosis-associated SVT; 18 were excluded because they had Budd–Chiari syndrome. Table 1 shows the baseline characteristics of the 149 included in the study. The most frequent etiology of cirrhosis was viral (55.7% of patients), followed by alcoholic (26.8%). At the time of their SVT diagnosis most patients were in Child–Pugh classes A and B. Fifty-six (37.6%) were on prophylactic medication or had undergone endoscopic treatment for variceal bleeding (21 were taking beta-blockers, 30 had received endoscopic treatment, and 5 had received both types of treatment). HCC was diagnosed in 26.2% of patients. The diagnosis of SVT was incidental in half of the cirrhotic patients. The most common clinical presentations were abdominal pain (31% of patients) and portal hypertensive bleeding (9.5%).

**Table 1:** Baseline characteristics of the study cohort

	Cirrhotic patients with SVT (*n*=149)
*Demographic characteristics*	
Age (years), median (IQR)	59 (50–68)
Men, *n/N* (%)	106/149 (71.1%)
Caucasian ethnicity, *n/N* (%)	120/149 (80.5%)
Asian ethnicity, *n/N* (%)	26/149 (17.5%)
Personal history of VTE, *n/N* (%)	9/146 (6.2%)
Family history of VTE, *n/N* (%)	7/144 (4.9%)
*Clinical presentation*	
Asymptomatic, *n/N* (%)	74/148 (50.0%)
Abdominal pain, *n/N* (%)	46/148 (31.1%)
Gastrointestinal bleeding (including hematemesis, hematochezia, melena), *n/N* (%)	14/148 (9.5%)
Fever, *n/N* (%)	10/148 (6.8%)
Nausea or vomiting, *n/N* (%)	6/148 (4.1%)
Anorexia, *n/N* (%)	3/148 (2.0%)
Diarrhea, *n/N* (%)	4/148 (2.7%)
Delay between symptom onset and diagnosis (days)^a^, median (IQR)	10 (4–20.5)
*Diagnostic methods*	
Computed tomography, *n/N* (%)	76/149 (51.0%)
Ultrasound, *n/N* (%)	61/149 (40.9%)
Others (magnetic resonance imaging or angiography), *n/N* (%)	12/149 (8.1%)
*Veins involved*	
Portal vein, *n/N* (%)	131/149 (87.9%)
Mesenteric veins, *n/N* (%)	51/149 (34.2%)
Splenic vein, *n/N* (%)	25/149 (16.8%)
Multiple veins, *n/N* (%)	46/149 (30.9%)
Collateralization at hepatic hilum	6/149 (4%)
*Comorbidities*	
Solid cancer, *n/N* (%)^b^	42/149 (28.2%)
Abdominal inflammation-infection, *n/N* (%)	4/149 (2.7%)
Recent abdominal surgery, *n/N* (%)	3/149 (2.0%)
*Severity of liver disease*	
Child–Pugh classification	
Median (IQR)	7 (5–8)
Class A (5–6 points)	59/127 (46.5%)
Class B (7–9 points)	55/127 (43.3%)
Class C (10–15 points)	13/127 (10.2%)
Serum total bilirubin	
<2 mg/dL	78/127 (61.4%)
2–3 mg/dL	23/127 (18.1%)
>3 mg/dL	26/127 (20.5%)
Serum albumin	
>3.5 g/dL	64/127 (50.4%)
2.8–3.5 g/dL	49/127 (38.6%)
<2.8 g/dL	14/127 (11.0%)
INR	
<1.7	115/127 (90.6%)
1.7–2.3	10/127 (7.9%)
>2.3	2/127 (1.6%)
Ascites	
No	77/127 (60.6%)
Mild	31/127 (24.4%)
Moderate	19/127 (15.0%)
Hepatic encephalopathy	
No	115/127 (90.6%)
Grade I–II	8/127 (6.3%)
Grade III–IV	4/127 (3.2%)
MELD score, median (IQR)	12 (10–16)
Etiology of liver cirrhosis	
Viral, *n/N* (%)	83/149 (55.7%)
Alcoholic, *n/N* (%)	40/149 (26.8%)
Mixed (viral and alcoholic), *n/N* (%)	4/149 (2.7%)
Cryptogenic, *n/N* (%)	12/149 (8.1%)
Other, *n/N* (%)	10/149 (6.7%)
Esophageal varices, *n/N* (%)	104/135 (77.0%)
Prophylaxis with beta-blockers, *n/N* (%)	26/104 (25.0%)
Endoscopic treatment, *n/N* (%)	35/104 (33.7%)
*Genetic mutations*	
JAK2 V617F mutation, *n/N* tested (total %)	1/32 (3.1%)
Prothrombin G20210A mutation, *n/N* tested (total %)	7/64 (10.9%)
Factor V Leiden mutation, *n/N* tested (total %)	7/71 (9.9%)
*Other laboratory test results*	
Anemia (hemoglobin ≤10 g/dL), *n/N* (%)	39/142 (27.5%)
Thrombocytopenia (platelet count ≤100 × 10^3^/mm^3^, *n/N* (%)	96/145 (66.2%)
Creatinine >1.5 mg/dL, *n/N* (%)	14/132 (10.6%)

*IQR* interquartile range, *SVT* splanchnic vein thrombosis, *VTE* venous thromboembolism

^a^Calculated only in patients with symptomatic onset

^b^Hepatocellular carcinoma in 39 of 42 oncological patients

The portal vein was the most common site of thrombosis, with 131/149 patients affected (87.9%), followed by the mesenteric veins (superior mesenteric thrombosis in 34%, inferior mesenteric vein thrombosis in 5%), and SVT occurred at multiple sites in 31% of cases. The distribution of the sites of thrombosis and their mode of presentation did not differ by liver disease severity in terms of Child–Pugh class (Table 2), not even after patients with HCC had been excluded from the analysis (data not shown).

**Table 2:** Baseline characteristics of the population by stage of liver cirrhosis

	Child–Pugh A (*n*=59)	Child–Pugh B (*n*=55)	Child–Pugh C (*n*=13)
*Demographic characteristics*			
Age (years), median (IQR)	56 (50–69)	58 (48–67)	61 (52–71)
Men, *n* (%)	42/59 (71.2%)	40/55 (72.7%)	8/13 (61.5%)
Asian ethnicity, *n* (%)	14/59 (23.7%)	11/55 (20.0%)	1/13 (7.7%)
Personal history of VTE, *n* (%)	2/58 (3.5%)	5/55 (9.1%)	1/11 (9.1%)
Family history of VTE, *n* (%)	6/59 (10.2%)	1/54 (1.9%)	0/10 (0%)
*Clinical presentation*			
Asymptomatic, *n* (%)	32/59 (54.2%)	28/55 (50.9%)	7/12 (58.3%)
Abdominal pain, *n* (%)	19/59 (32.2%)^a^	16/55 (29.1%)	0/12 (0%)^a^
Gastrointestinal bleeding (including hematemesis, hematochezia, melena), *n* (%)	7/59 (11.9%)	4/55 (7.3%)	1/12 (8.3%)
Delay between symptom onset and diagnosis (days), median (IQR)	10 (3–30)	7 (4–20)	18 (11–21)
*Veins involved*			
Portal vein, *n* (%)	51/59 (86.4%)	48/55 (87.3%)	12/13 (92.3%)
Mesenteric veins, *n* (%)	18/59 (30.5%)	21/55 (38.2%)	2/13 (15.4%)
Splenic vein, *n* (%)	11/59 (18.6%)	8/55 (14.6%)	1/13 (7.7%)
Multiple veins, *n* (%)	17/59 (28.8%)	17/55 (30.9%)	2/13 (15.4%)
*Comorbidities*			
Hepatic cirrhosis as sole risk factor, *n* (%)	43/59 (72.9%)	38/55 (69.1%)	9/13 (69.2%)
Hepatocellular carcinoma, *n* (%)	11/59 (18.6%)	15/55 (27.3%)	4/13 (30.8%)
Abdominal inflammation-infection, *n* (%)	4/59 (6.8%)	0/55 (0%)	0/13 (0%)
Recent abdominal surgery, *n* (%)	1/59 (1.7%)	2/55 (3.6%)	0/13 (0%)
*Severity of liver disease*			
Esophageal varices, *n* (%)	45/59 (76.3%)	42/54 (77.8%)	9/13 (69.2%)
Child–Pugh score, median (IQR)	5 (5–6)^b,c^	8 (7–8)^b,d^	10 (10–11)^d,c^
MELD score, median (IQR)	10 (9–11)^b,c^	14 (12–17)^b,d^	21 (19–26)^d,c^
*Therapeutic strategies started during the acute phase*			
No treatment, *n* (%)	23/59 (39.0%)	20/55 (36.4%)	8/13 (61.5%)
Any anticoagulant, *n* (%) treatment duration, median (IQR)	36/59 (61.0%), 14.5 months (5.6–24)^e^	35/55 (63.6%), 6 months (3–11)^e^	4/13 (30.8%), 4.2 months (0.8–8)
Parenteral treatment only (UFH, LMWH, or fondaparinux), *n* (%) treatment duration, median (IQR)	25/59 (42.4%)^a^, 10.1 months (5–23)^e^	19/55 (34.5%), 4 months (2.2–6)^e^	1/13 (7.7%)^e^, 7 months (7–7)
Vitamin K antagonist, *n* (%) treatment duration, median (IQR)	11/59 (18.6%), 19.5 months (6–24)	16/55 (29.1%), 6 months (4–24)	3/13 (23.1%), 1.4 months (0.1–9)

*IQR* interquartile range, *LMWH* low-molecular-weight heparin, *UFH* unfractionated heparin, *VTE* venous thromboembolism

The Child–Pugh score was not available for 22 patients

^a^*P* < 0.05 for the comparison between Child–Pugh class A and C

^b^*P* < 0.01 for the comparison between Child–Pugh class A and B

^c^*P* < 0.01 for the comparison between Child–Pugh class A and C

^d^*P* < 0.01 for the comparison between Child–Pugh class B and C

^e^*P* < 0.05 for the comparison between Child–Pugh class A and B

Anticoagulant treatment was administered during the acute phase to 92 patients (61.7%): 6 (4.0%) were given unfractionated heparin, 84 (56.4%) LMWH or fondaparinux, and 32 (21.5%) were started on VKA. Six patients underwent surgical or radiological procedures for thrombosis treatment. Sixty patients (40.3%) continued with parenteral anticoagulation alone, with various dosages and regimens. The anticoagulation treatments lasted a median 6.5 months (IQR: 3.9–22.5); their duration was shorter for patients given only parenteral treatments (6 months; IQR: 3–16) than for those who received oral anticoagulants (9.5 months; IQR: 4–24; *P* = 0.1752). Factors associated with the use of anticoagulant drugs were the local extent of thrombosis and the presence of mesenteric vein thrombosis (Table 3).

**Table 3:** Baseline characteristics of the population by therapeutic approach

	No treatment (*n*=56)	Anticoagulation (*n*=92)	*P-*value
*Demographic characteristics*			
Age (years), median (IQR)	56 (49–65.5)	61 (52–69)	0.138
Men, *n/N* (%)	42/56 (75.0%)	64/92 (69.6%)	0.477
Asian ethnicity, *n/N* (%)	16/56 (28.6%)	10/92 (10.9%)	0.006
Personal history of VTE, *n/N* (%)	2/54 (3.7%)	7/91 (7.7%)	0.485
Family history of VTE, *n/N* (%)	2/53 (3.8%)	5/90 (5.6%)	1
*Clinical presentation*			
Asymptomatic, *n/N* (%)	32/55 (58.2%)	41/92 (44.6%)	0.110
Abdominal pain, *n/N* (%)	13/55 (23.6%)	33/92 (35.9%)	0.122
Gastrointestinal bleeding (including hematemesis, hematochezia, melena), *n/N* (%)	7/55 (12.7%)	7/92 (7.6%)	0.306
Delay between symptom onset and diagnosis (days)*, median (IQR)	16.5 (8.5–30)	7 (3–20)	0.082
*Veins involved*			
Portal vein, *n/N* (%)	51/56 (91.1%)	79/92 (85.9%)	0.348
Mesenteric veins, *n/N* (%)	12/56 (21.4%)	39/92 (42.4%)	0.009
Splenic vein, *n/N* (%)	6/56 (10.7%)	19/92 (20.7%)	0.118
Multiple veins, *n/N* (%)	10/56 (17.9%)	36/92 (39.1%)	0.007
*Comorbidities*			
Hepatic cirrhosis as sole risk factor, *n/N* (%)	38/56 (67.9%)	63/92 (68.5%)	0.920
Hepatocellular carcinoma, *n/N* (%)	17/56 (30.4%)	22/92 (23.9%)	0.388
Abdominal inflammation infection, *n/N* (%)	1/56 (1.8%)	3/92 (3.3%)	1
Recent abdominal surgery, *n/N* (%)	0/56 (0%)	3/92 (3.3%)	0.290
*Severity of liver disease*			
Child–Pugh score, median (IQR)	7 (5–8)	7 (5–8)	0.517
MELD score, median (IQR)	12 (10–17)	12 (10–15)	0.527
Esophageal varices, *n/N* (%)	46/55 (83.6%)	58/79 (73.4%)	0.163
*Other laboratory test results*			
Anemia (hemoglobin ≤10 g/dL), *n/N* (%)	17/53 (32.1%)	21/88 (23.9%)	0.287
Thrombocytopenia (platelet count ≤100 × 10^3^/mm^3^, *n/N* (%)	35/55 (63.6%)	60/89 (67.4%)	0.642
Creatinine >1.5 mg/dL, *n/N* (%)	4/55 (7.3%)	10/76 (13.2%)	0.393

*IQR* interquartile range, *VTE* venous thromboembolism

One patient was excluded from this analysis because she had only an interventional procedure, without anticoagulant treatment

*Calculated only in patients with symptomatic onset

The total duration of the follow-up for cirrhotic SVT patients was 194.01 patient-years, with a median duration of 1.7 years (IQR: 0.5–2 years). Four patients (2.7%) were lost to follow-up.

### Major bleeding episodes and thrombotic events

**Fig. 1:**
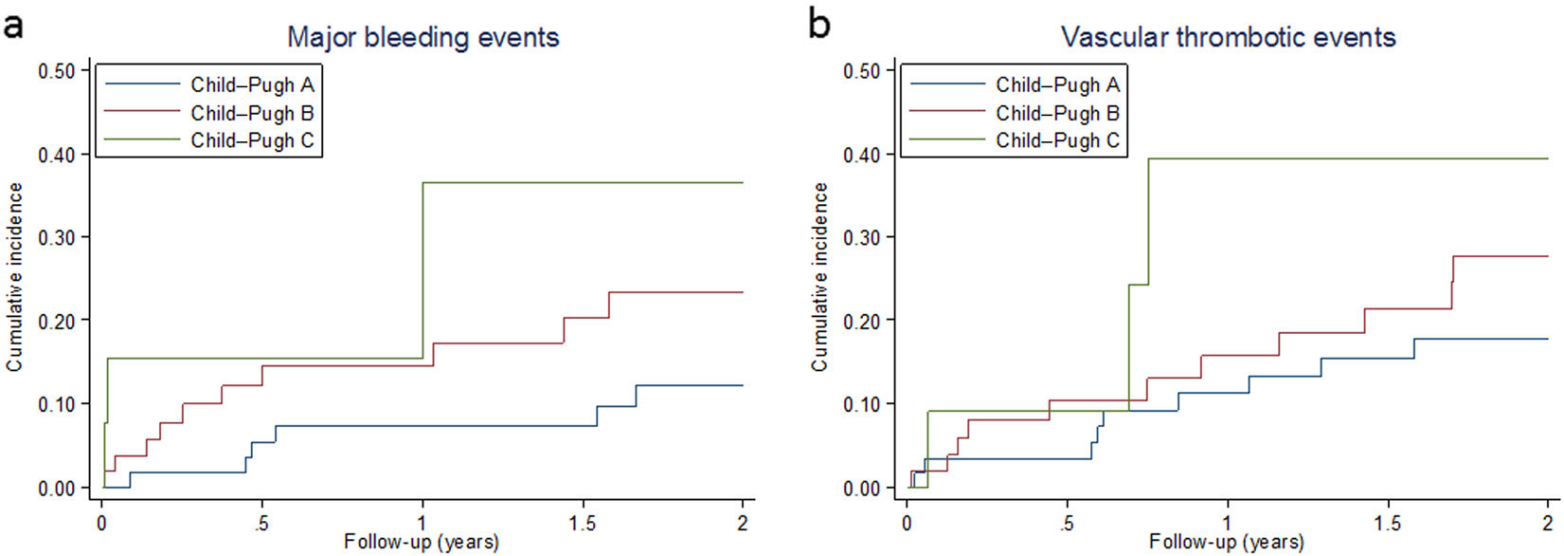
Kaplan–Meier curve for the incidence of major bleeding events (**a**), and vascular thrombotic events (**b**)

During the follow-up, there were 19 major bleeding episodes, meaning an overall incidence of 9.8 per 100 patient-years (95% CI: 6.2–15.4). Their incidence was higher for more severe liver disease, being 6.8 (95% CI: 3.0–15.1), 15.4 (95% CI: 8.3–28.6), and 33 (95% CI: 10.6–102.4) in Child–Pugh classes A, B, and C, respectively. The cumulative incidence of major bleeding episodes by Child–Pugh class is shown in Fig. 1a.

Sixteen (84%) major bleeding episodes were gastrointestinal: 10 from esophageal varices (one of them fatal), and 6 from other gastrointestinal sites; the other three were intracranial or intraspinal hemorrhages. The incidence of major bleeding episodes among the patients given anticoagulants was higher during the treatment than after its discontinuation (10.2 [95% CI: 5.3–19.6] versus 3.1 [95% CI: 0.4–22.3] per 100 patient-years), and no different from the incidence seen in the untreated group (12.3 [95% CI: 5.9–25.8] per 100 patient-years). On multivariable analysis, only time on treatment (HR% CI: 0.83 [0.69–0.99], *P* = 0.038) was independently associated with the risk of bleeding episodes.

There were 25 thrombotic events, with an incidence of 12.9 per 100 patient-years (95% CI: 8.7–19.1): 23 events (92%) in the splanchnic venous system, 1 acute coronary syndrome, and 1 vascular death. Patients with more severe liver disease were at higher risk of recurrent vascular events during the follow-up, with an incidence of 10.1 (95% CI: 5.3–19.5), 16.9 (95% CI: 9.4–30.6), and 33 (95% CI: 10.6–102.40) per 100 patient-years for patients in Child–Pugh classes A, B, and C, respectively. The cumulative incidence of thrombotic events by Child–Pugh class is shown in Fig. 1b. The incidence of thrombotic events in patients receiving anticoagulant treatment was 9.1 per 100 patient-years (95% CI: 4.5–18.1), and it rose to 18.9 (95% CI: 8.5–42.0) in those who had discontinued the treatment. The time elapsed between discontinuation of anticoagulation therapy and the event was a median of 5 months (IQR: 2.2–9.9, range: 1.5–14.5). In the subgroup of patients, who had never received anticoagulation, the incidence of thrombotic events was 17.6 per 100 patient-year (95% CI: 9.5–32.7). On multivariable analysis, only time on treatment (HR% CI: 0.85 [0.76–0.96], *P* = 0.006) was independently associated with the reduction in the risk of recurrent thrombotic events.

### Mortality

**Fig. 2:**
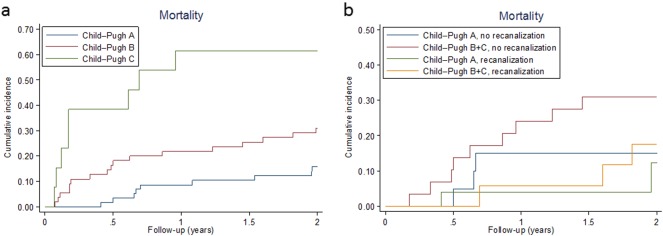
Kaplan–Meier curve for mortality by Child–Pugh class (**a**), and response to anticoagulation (**b**)

Considering the total cohort, there were 42 deaths altogether during the follow-up, with an incidence of 17.5 per 100 patient-years (95% CI: 12.9–23.7), and the risk of death was higher in patients with more advanced liver disease (Fig. 2a). Overall, there were three bleeding-related deaths (two from esophageal varices, one of them while the patient was on anticoagulant treatment), and one vascular death (sudden cardiac arrest in a patient not taking anticoagulants). Twenty-five deaths were associated with underlying diseases (12 were due to cancer progression, 8 due to liver failure, and 5 due to both), four were due to other causes, and for nine the cause could not be ascertained. On multivariable analysis, ascites, a personal history of venous thromboembolism, and HCC correlated with a higher mortality risk (Table 4). Nine patients (6%) underwent TIPS placement. Among the 138 patients with information available about other outcomes, 16 patients (11.6%) underwent liver transplantation.

**Table 4:** Factors associated with mortality on multivariable analysis

Variable	Hazard ratio (95% CI)	*P*-value
Personal history of venous thromboembolism	14.73 (2.11–102.83)	0.007
Hepatocellular carcinoma	6.24 (2.45–15.85)	< 0.001
Ascites	5.65 (1.92–16.64)	0.002

Only variables associated with *P*-values <0.05 are shown. All other variables were removed during the backward stepwise procedure

### Vessel recanalization during follow-up

In the subgroup of 98 patients with available follow-up imaging enabling an analysis of their SVT, 61 underwent imaging more than once (median 2, range 1–5). The timing of follow-up imaging tests ranged from 0.13 to 24 months. During the follow-up, 47 (48.0%) patients had a total or partial resolution of thrombosis. The median time to recanalization was 6.16 months (IQR: 4.3–12.82).

Anticoagulant treatment during the acute phase increased the recanalization rate by comparison with no anticoagulation (54.8% versus 36.1%). On multivariable analysis, anticoagulant treatment was associated with a better imaging outcome during the follow-up [HR (95% CI): 3.33 (1.42–7.84), *P* = 0.006]. Interestingly, the extent of thrombosis with involvement of the portal vein and superior mesenteric veins was not associated with a higher risk of local recurrence. Mortality rates were 6.8 per 100 patient-years (95% CI: 3.1–15.1) in patients with partial or complete recanalization during the follow-up and 15.4 per 100 patient-years (95% CI: 8.9–26.5) in those with stable or progressing thrombi (*P* = 0.092), and the impact on survival was greater in cirrhotics with more advanced liver disease (Child–Pugh B and C versus A), as shown in Fig. 2b.

## Discussion

The results in this large, prospective cohort of patients with cirrhosis-associated SVT shed light on the long-term clinical outcomes of SVT in the cirrhotic population in real-world clinical practice. The baseline characteristics and presentations of SVT were similar to those described in previously published cohorts, the portal vein being the most common site of SVT^3,7,8,12–16^, and the incidental finding of SVT accounting for nearly 50% of cases^17^. These proportions did not differ by Child–Pugh class, confirming that the risk of PVT correlates more with portal hypertension than with liver disease severity^18^. In the cohort described here, the decision to administer anticoagulants during the acute phase seemed to be clinically driven by the extent of thrombosis and the involvement of the superior mesenteric vein, with the associated risk of intestinal infarction.

In the general population, the clinical benefit of anticoagulation must be weighed against the bleeding risk, and patients with cirrhosis are no exception to this rule. During the follow-up, the risk of recurrent thrombotic events was associated with the severity of patients’ liver disease (in terms of Child–Pugh class), suggesting that an imbalance in the factor VIII/protein C ratio, and therefore in thrombin generation^19^, and a more severe portal hypertension^4,18^ are risk factors for SVT in the cirrhotic population. Interestingly, the risk of recurrent SVT was identical for patients left untreated and those who discontinued anticoagulation. This finding is in keeping with reports of 40% of cases of PVT recurring after treatment^7^, and supports the recommendation that anticoagulation be prolonged beyond 6 months. In the light of the high rates of recurrence observed in this cohort, an extended anticoagulant treatment may be considered for all patients who have safely completed the first 6 months of therapy. However, this approach should be assessed in specifically designed studies.

The risk of major bleeding episodes—half of which stemmed from esophageal varices in our cohort (10/19)—was also associated with liver disease severity, but was much the same for patients with and without anticoagulant treatment. It is worth noting that, although more bleeding episodes occurred in patients taking anticoagulants than in those who had discontinued the treatment, most of the former cases were not related to portal hypertension. On multivariable analysis, a platelet count of less than 50,000 per mm^3^ did not correlate with the risk of bleeding while on anticoagulants in our cohort, in contrast with the findings of Delgado et al^8^. After the treatment (which lasted a median of 6 months) was discontinued, the risk of bleeding dropped significantly (from 10.2 to 3.1 per 100 patient-years), probably due to the resolution of the PVT (in 90% of cases) among patients who responded to anticoagulation. The risk of major and minor bleeding episodes posed by anticoagulation in cirrhosis-associated PVT may be set against the fact that PVT per se increases the severity of any variceal bleeding. In two other cohorts^6,8^, resolution of thrombosis was shown to reduce the pressure on esophageal varices^20^ and to correlate with fewer bleeding episodes from esophageal varices, and it correlated independently with fewer decompensations in cirrhotics prophylactically treated in a randomized controlled trial to prevent PVT^21^.

Although any abdominal imaging during the follow-up was left to the discretion of the attending clinicians, 98 patients had at least one imaging test that could be reviewed to establish the efficacy of anticoagulation treatment in restoring vessel patency (which occurred in 54.8% of cases). To date, nine series have been published regarding similar anticoagulation regimens and reporting similar response rates. In cirrhotic patients treated with VKA, the response rate ranged from 42% to 82%^3,13,22^. The median duration of anticoagulation treatment was at least 6 months in most of the published studies, but it was prolonged for at least 12 months in non-responders and partial responders in only three studies^6,7,13^. In our cohort, about one in three patients obtained a good vessel recanalization without taking anticoagulants, thus confirming the data from Luca et al.^23^ on the natural history of untreated PVT. As for the time to recanalization, the variable timing of imaging undertaken during the follow-up prevents us from drawing any conclusions on the present cohort.

The impact of SVT, and PVT in particular, on the natural history of liver cirrhosis is still under debate. A recent paper from Nery et al. suggested that untreated PVT may undergo spontaneous recanalization without significantly deteriorating the course of cirrhosis^22^, but Luca et al. found a (statistically insignificant) increase in mortality in the group of patients who had stable or progressing thrombi (39.1% versus 15.8%)^23^. In our cohort, the annualized mortality rates were higher in patients with cirrhosis with no evidence of vessel recanalization during the follow-up, and this effect on survival was only seen in patients with more advanced liver disease (Child–Pugh B and C), not in Child–Pugh A patients. This confirms the picture seen in the cohort described by Nery et al., in which all patients had very stable liver function and the persistence of PVT did not affect the natural history of cirrhosis. This might not be the case in more advanced liver disease^5^, but larger studies will be needed to confirm as much.

The present study has several limitations due to its observational design. The therapeutic approach to PVT in patients with cirrhosis was left entirely to the discretion of participating clinicians, because one of our main aims was to describe current management strategies adopted for SVT. This approach also enabled us to describe the clinical and natural history of this disease in untreated patients too, and to shed light on the impact of persistent PVT on the natural history of cirrhosis in patients with decompensated liver disease. On the other hand, the study design used here is unsuitable for the purpose of validating one treatment strategy as opposed to another. To the best of our knowledge, this is currently the largest prospective cohort study on patients with cirrhosis-associated PVT with a sufficiently long follow-up.

Another important limitation concerns the fact that any follow-up imaging of PVT was not done according to a strict protocol. The timing of follow-up imaging differed because it was planned on a case-by-case basis by the treating physicians. That said, our analysis of the subset of patients with follow-up imaging and of the methods employed produced results similar to those reported by Herweh et al.^11^, when they analyzed the frequency and timing of recanalization after cerebral vein and sinus thrombosis. Despite the possible bias in this present subset, the response rate, and the OR for recanalization with and without anticoagulants were very similar to those of previously published studies^24^, so our clinical interpretation of the outcomes is probably valid.

To reduce the limitations associated with the study’s observational design, the coordinating center routinely monitored all data provided in the electronic case report forms, and all clinical outcomes were confirmed by a central review committee.

In conclusion, the results of this study confirm that patients with cirrhosis are at very high risk of recurrent thrombotic events, and this risk is associated with the severity of their liver disease. The use of anticoagulants did not increase the bleeding risk in our cohort, and the long-term decrease in the bleeding rates was possibly related to recanalization of the portal vein after treatment. Partial or total resolution of thrombosis may also reduce the mortality risk in patients with more advanced liver disease.

## Study Highlights

### What is current knowledge

- Splanchnic (and particularly portal) vein thrombosis (SVT) is a common complication in patients with end-stage liver disease.
- There is no consensus on whether this complication correlates with a higher mortality, so anticoagulation therapy is still not always prescribed.

### What is new here

- Patients with portal vein thrombosis are at high risk of recurrent thrombotic episodes in the splanchnic veins, which can be significantly reduced by anticoagulation therapy.
- Patients given anticoagulants for SVT experienced fewer thrombotic events during the treatment, and fewer bleeding episodes afterwards, than patients left untreated.
- Patients with decompensated liver disease (Child–Pugh B-C) may benefit particularly from anticoagulation because survival was better for those whose thrombosis improved.

## Electronic supplementary material

Supplementary File: List of participating centers
