# Supplementary material for: Long-Term Outcome of Splanchnic Vein Thrombosis in Cirrhosis
Source: Clin Transl Gastroenterol. 2018 Aug 15;9(8):176. doi: 10.1038/s41424-018-0043-2 (PMC6092393; doi:10.1038/s41424-018-0043-2)
Supplement: Supplementary file 1 — Supplementary File: List of participating centers [file 41424_2018_43_MOESM1_ESM.docx]

**LONG-TERM OUTCOME OF SPLANCHNIC VEIN THROMBOSIS IN CIRRHOSIS**

**Authors:** Marco Senzolo^1^, Nicoletta Riva^2^, Francesco Dentali^2^, Kryssia Rodriguez-Castro^1^, Maria Teresa Sartori^9^, Soo-Mee Bang^3^, Ida Martinelli^4^, Sam Schulman^5^, Adriano Alatri^6^, Jan Beyer-Westendorf^7^, Matteo Nicola Dario Di Minno^8^, Walter Ageno^2^, for the IRSVT study investigators

**Supplementary File: List of participating centers**

1. Department of Internal Medicine, Seoul National University, South Korea
2. Multivisceral Transplant Unit, Gastroenterology, University Hospital of Padova and Clinical Medicine I, University Hospital of Padova, Italy
3. IRCCS Casa Sollievo della Sofferenza, S. Giovanni Rotondo, and A.O.R.N. Ospedale Cardarelli, Napoli, Italy
4. Center for Vascular Medicine and Department of Medicine III, Division of Angiology, University Hospital “Carl Gustav Carus” Dresden, Germany
5. Center for Hemorrhagic and Thrombotic Diseases, University Hospital, Udine, Italy
6. Department of Clinical and Experimental Medicine, University of Insubria, Varese, Italy
7. Unit of cell and molecular biology in cardiovascular diseases, Centro Cardiologico Monzino, IRCCS, Milan, Italy
8. Department of Medicine, McMaster University, Hamilton, Ontario, Canada
9. Thrombosis Center, Galliera Hospital, Genoa, Italy
10. Cattedra ed UO di Ematologia con Trapianto, Policlinico Universitario di Palermo, Italy
11. Haemophilia Center, Azienda Ospedaliera Pugliese-Ciaccio, Catanzaro, Italy
12. Thrombosis Center, Careggi Hospital, Florence, Italy
13. Vascular Medicine and Haemostasis, University of Leuven, Leuven, Belgium
14. Hemophilia and Thrombosis Center, Fondazione IRCCS Ca’ Granda - Ospedale Maggiore Policlinico, Milan, Italy
15. University of Groningen, the Netherlands
16. Centro Emostasi e Trombosi, A.O. Istituti Ospitalieri di Cremona, Italy
17. Department of Internal Medicine, Pochon CHA University, Seoul, Korea
18. Hospital das Clínicas da Faculdade de Medicina, Universidade de Sao Paulo, Brazil
19. Department of Internal and Vascular Medicine, University of Perugia, Ospedale S. Maria della Misericordia, Perugia, Italy
20. Ospedale di Faenza, Italy
21. Department of Internal Medicine, University of Genoa, Italy
22. Institute of Hematology, Catholic University, Rome, Italy
23. Department of Clinical Medicine, University Hospital of Sassari, Italy
24. UOSD di Ematologia e Malattie Trombotiche Ospedale San Giovanni Bosco - Torino
25. Department of Medicine I, Busto Arsizio Hospital, Italy
26. Department of Medical, Oral, and Biotechnological Sciences, University G. D’Annunzio, Chieti, Italy
27. Hematology-Medical Oncology Division, Weill-Cornell Medical College
28. Unité dei Médecine Interne et Pathologie Vasculaire, Université Denis Diderot, Paris, France
29. Coagulation Unit, Wolfson Medical Center, Israel
30. Hematology Division, S. Gerardo Hospital, Monza, Italy
31. Leeds General Infirmary, UK
